# COVID-19 waves: variant dynamics and control

**DOI:** 10.1038/s41598-022-13371-2

**Published:** 2022-06-04

**Authors:** Abhishek Dutta

**Affiliations:** Department of Electrical and Computer Engineering, University of Connecticut, Storrs, CT 06269 USA

**Keywords:** Computational biology and bioinformatics, Systems biology, Diseases

## Abstract

The waves of COVID-19 infections driven by its variants continue to nullify the success we achieved through efficacious vaccines, social restrictions, testing and quarantine policies. This paper models the two major variants-driven waves by two sets of susceptible-infected-quarantined-recovered-vaccinated-deceased coupled dynamics that are modulated by the three main interventions: vaccination, quarantine and restrictions. This $SI^2Q^2R^2VD$ system is used to demonstrate that the second major novel coronavirus wave in the US is caused by the delta variant and the corresponding rapid surge in infectious cases is driven by the unvaccinated pool of the populace. Next, a feedback control based planned vaccination strategy is derived and is shown to be able to suppress the surge in infections effectively.

## Introduction

The major waves of COVID-19 infections, after the initial onset, are largely caused by the emerging variants of concern^1^. They prolong the persistence of infections inflicting loss to human lives, economies and continue to strain the public health infrastructure^2^. The development of highly efficacious vaccines against COVID-19 and its variants have indeed proven to be effective in averting symptomatic infections and illnesses^3^. However, despite the availability of these vaccines and repurposed antiviral therapies^4^, the recent COVID-19 wave of infections have wreaked havoc. Recent studies have analyzed the second surge behavior of COVID-19 in the US states^5^ and associated mortality in the US and Europe^6^. The alarming rise of novel coronavirus variants necessitates genomic surveillance for early anticipation and initiation of mitigation strategies to contain such outbreaks^7^ . Problems connected with vaccine hesitancy and the availability and distribution of vaccines in the poorest countries must be countered with systematic vaccination policies and plans^8,9^. Therefore, it is important to mathematically and objectively analyze the underlying factors that fuel these subsequent surges in infections and possible mitigation strategies.

Nonlinear dynamical models involving some combination of susceptible/S, infected/I, recovered/R together with some additional compartments have been utilized to predict the initial evolution of this pandemic^10,11^. Early mitigation strategies based on feedback control were developed to achieve a decay in infections through planned interventions in terms of levels of social restrictions^12^ and number of tests and quarantine^13^. However, once efficacious vaccines were being developed and administered, these models needed to be adapted to include the vaccination rates and states. So, age structured vaccinated/V compartments were added to evaluate age group prioritized vaccination strategies^14^ whereas the effect of two vaccine doses was modeled with two V states^15^. Two additional states of quarantined/Q and deceased/D were included followed by an ensemble Kalman filter design to estimate the evolution of infections^16^. A simple SIR model plus exposed/E state with prophylactic and therapeutic interventions was considered to analyze the stability of the pandemic^17^. A similar model was simulated with stochastic transmission and recovery rates^18^ and analyzed in a discrete time setting with auto-regressive transmission rate^19^.

Early optimal control based vaccine administration by minimizing number of infections over a time horizon have been formulated with constraints on daily vaccination rate^20^. Optimal control strategy based on age-structured administration^21^ and together with regional dynamics^22^ were also explored. Optimal vaccination polices that aim to decrease the reproduction number were examined^23^ together with the addition of plasma transfusion as control variable^24^. However, this body of work did not consider the added complexities posed by the emergent variants of SARS-CoV-2. Some of the recent work that consider variants include multiple transmissions rates corresponding to the different strains^15^ and use multiple sets of SIRV states to track the evolution of the respective viral infection strains and analyze stability^25^. Optimal control has recently been applied to variant models to compute an effective restriction policy^26,27^.

In spite of these developments, what is lacking is a systemic analysis of the major waves, their underlying cause and an effective mitigation policy. In this paper, a $SI^2Q^2R^2VD$ system is introduced to explain the evolution of the two variants and establish the delta variant as the driver of the second major wave in the US (peaking in September, 2021). The variant-specific rates of transmission, along side recovery, mortality, test and quarantine, vaccination rates are all learnt from reported data^28^ for the US during January 22, 2020 to November 30, 2021 by gradient-free stochastic optimization. From here, the unvaccinated pool of people driving the second wave of surge in infections is established. Finally, feedback control based planned vaccination strategies are shown to be able to suppress the number of cases and mortality along with the duration of the pandemic.

## Methods

The initial havoc caused by the novel coronavirus in terms of mortality, mental and economic health in the year 2020 was coming under control by the development and administration of efficacious vaccines through early 2021, until the devastation caused by the emerging variants of interest. Therefore, the epidemic models, controls and analysis which revolve around a single strain need to be adapted to explain the recurrent waves associated with the dominant strains.

### Variant dynamics

**Figure 1:**
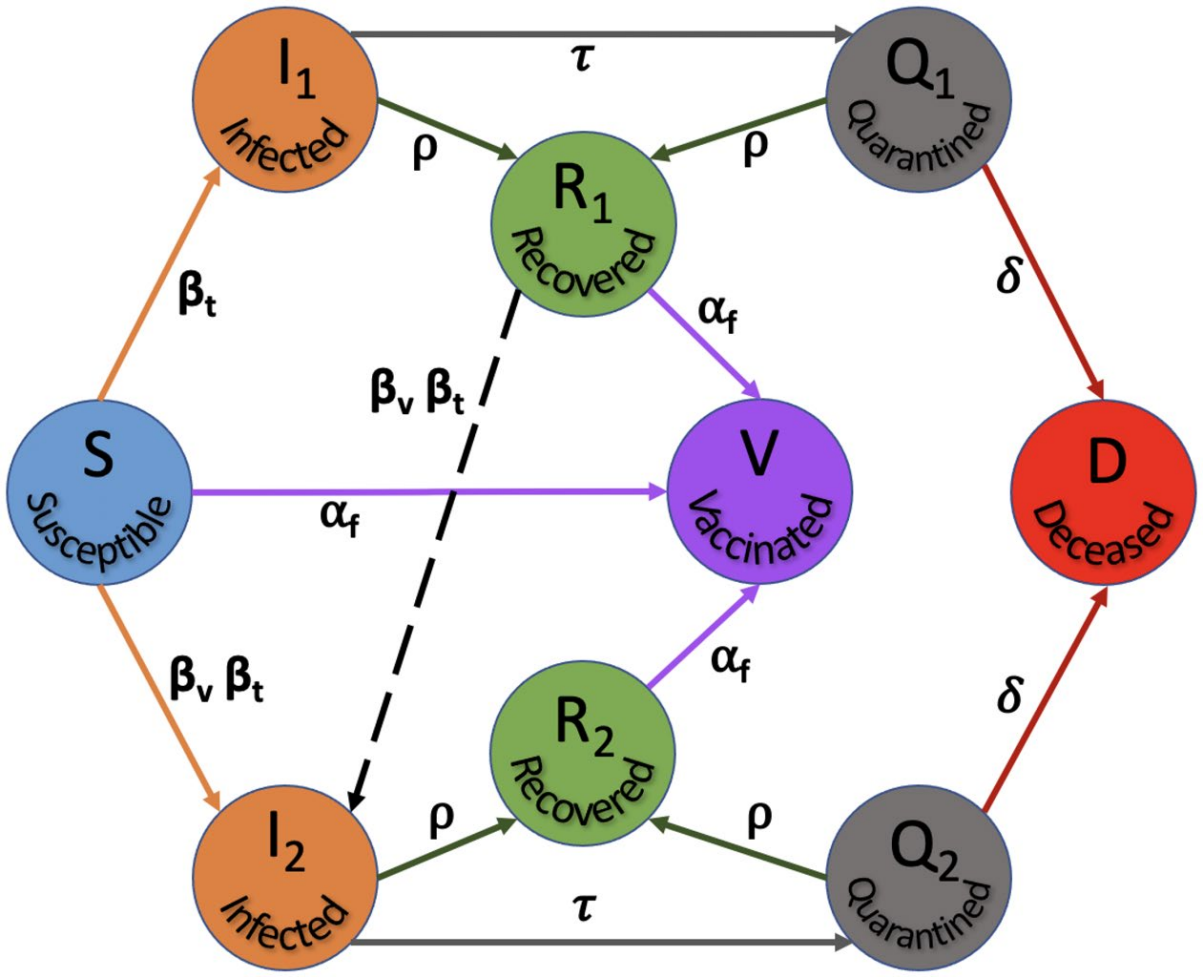
The $SI^2Q^2R^2VD$ dynamics governed by $\beta _t,\beta _v,\alpha _f,\tau ,\rho ,\delta$ rate parameters.

Here, the $SI^2Q^2R^2VD$ nonlinear coupled dynamical system is introduced to model the two dominant variants and associated waves in the US, see Fig. 1. The susceptible/S can be infected initially by the dominant strain with a rate of transmission $\beta _t$ and later with the other dominant variant with a multiplier $\beta _v$ to accommodate more/less infectious nature of the second variant, to end up in the infected pools $I_1, I_2$ respectively. These infected are actively tested and quarantined with rate $\tau$ and pushed to compartments $Q_1,Q_2$, respectively.

Meanwhile, the infected and quarantined populace can recover with rate $\rho$ to end up in $R_1,R_2$ corresponding to dominant strains 1, 2 respectively. Vaccines are actively administered at rate $\alpha _f$ to the susceptible and recovered, who then make up the vaccinated/V compartment. Those who succumb to the disease move from quarantine to deceased/D with mortality rate $\delta$. The key here is that this model accommodates the fact that people who recover from the alpha strain and remain unvaccinated can be infected by the second strain. The full $SI^2Q^2R^2VD$ system is formulated through equations (1)–(9), with *N* the total population.

1 $$\begin{aligned}&\dot{S}=-(1-\alpha _f)\frac{\beta _t S(I_1+I_2\beta _v)}{N}-\alpha _f S \end{aligned}$$

2 $$\begin{aligned}&\dot{I}_1=(1-\alpha _f)\frac{\beta _t SI_1}{N}-(\tau +\rho )I_1 \end{aligned}$$

3 $$\begin{aligned}&\dot{I}_2=(1-\alpha _f)\frac{\beta _t (S+R_1)I_2\beta _v}{N}-(\tau +\rho )I_2 \end{aligned}$$

4 $$\begin{aligned}&\dot{Q}_1=\tau I_1-(\rho +\delta ) Q_1 \end{aligned}$$

5 $$\begin{aligned}&\dot{Q}_2=\tau I_2-(\rho +\delta ) Q_2 \end{aligned}$$

6 $$\begin{aligned}&\dot{R}_1=\rho (I_1+Q_1)-\alpha _f R_1-(1-\alpha _f)\frac{\beta _t R_1 I_2\beta _v}{N} \end{aligned}$$

7 $$\begin{aligned}&\dot{R}_2=\rho (I_2+Q_2)-\alpha _f R_2 \end{aligned}$$

8 $$\begin{aligned}&\dot{V}=\alpha _f (S+R_1+R_2) \end{aligned}$$

9 $$\begin{aligned}&\dot{D}=\delta (Q_1+Q_2) \end{aligned}$$

Note that, the baseline rate of transmission $\beta _t$ needs to be time-varying to reflect continued gradual relaxation of social restrictions after imposing a severe lockdown, that can be accommodated by a sigmoid function of (18) with lower, upper limits of $\underline{\beta },\bar{\beta }$ respectively. Since the vaccines and delta variant were introduced in the middle of the pandemic, they can be modeled by step functions of (19) with *v*, *f* denoting the respective start of the delta variant and fully vaccinated individuals. The disease free equilibrium/DFE can be obtained by equating all the derivatives in (1)–(9) to the zero vector, to be

10 $$\begin{aligned} DFE\equiv \{S^0,I_1^0,I_2^0,Q_1^0,Q_2^0,R_1^0,R_2^0,V^0,D^0\}\in \mathbb {R}^9_+:I_1^0=I_2^0=Q_1^0=Q_2^0=0,S^0+R_1^0+R_2^0+V^0+D^0=N {,} \end{aligned}$$

where the superscript $\cdot ^0$ denotes the DFE. The time-varying rates of reproduction of the variants, $\mathcal R_t^0$ (alpha variant) during the first wave and $\mathcal R_t^v$ (delta variant) during the second wave, can be derived from (16) to be

11 $$\begin{aligned}&\mathcal R_t^0 = \frac{(1-\alpha _f)S\beta _t}{(\rho +\tau )N} {,}\end{aligned}$$

12 $$\begin{aligned}&\mathcal R_t^v = \frac{(1-\alpha _f)\beta _t(S+R_1)\beta _v}{(\rho +\tau )N} {,} \end{aligned}$$

with the overall time-varying rate of reproduction of the pandemic given by $\mathcal R_t=\max (\mathcal R_t^0,\mathcal R_t^v)$.

### Parameter tdentification

The $SI^2Q^2R^2VD$ system is modeled as per the set of coupled nonlinear differential equations of (1)–(9) with $I_1,Q_1,R_1$ and $I_2,Q_2,R_2$ corresponding to the two major variant-driven waves. The reported number of confirmed cases, recovered, fully vaccinated and deceased over the span of the pandemic are recorded in vectors $I_r,R_r,V_r,D_r$ respectively from the Johns Hopkins public repository^28^. The various underlying epidemic rate and intervention parameters are learnt from this data by solving the optimization problem of (13) that minimizes its difference to the model predictions,

13 $$\begin{aligned} \min _{\{a, b, v, \beta _v, \alpha _f, \tau , \rho , \delta \}\in \mathbb {R}^8_+}J=||\tau \int _0^T(I_1+I_2)dt_{|_d}-I_r||_2^2+||\int _0^{T_r}Rdt_{|_d}-R_r||_2^2+||\int _T^TVdt_{|_d}-V_r||_2^2+||\int _0^TDdt_{|_d}-D_r||_2^2 {,} \end{aligned}$$

constrained to the dynamics of the $SI^2Q^2R^2VD$ system (1)–(9), where the output of the integrals are computed daily ${\cdot |_d}$ and $||\cdot ||_2^2$ are the squared $l^2$ norm. The limit of integration for infections, deceased and fully vaccinated run through the span of the pandemic or the interval of analysis *T*. The lack of recovery data after $T_r$ is reflected by the corresponding upper limit. The minimization problem of (13) being constrained to the underlying nonlinear dynamics of the $SI^2Q^2R^2VD$ system of (1)–(9), makes it hard to compute the gradient of the cost function *J* analytically. Therefore, a derivative-free stochastic optimization technique based on Nelder and Mead^29^ based on evolving simplexes of solutions is used to find the best set of rate parameters $\underline{\beta },\bar{\beta },v,\beta _v,\alpha _f,\tau ,\rho ,\delta$, the algorithm follows:

Initialize: An $n+1$ dimensional simplex is formed around the seed value of $p=[\underline{\beta },\bar{\beta },v,\beta _v,\alpha _f,\tau ,\rho ,\delta ]$, with associated cost *J*(*p*) from (13). Here, the number of dimensions $n=8$.
Order: Evaluate the cost function at vertices by numerically integration and sort $J(p_1)\le \ldots J(p_n)\le J(p_{n+1})$ and compute the centroid $p_0=\frac{1}{n}\sum _1^n p_i$.
Terminate: If standard deviation of simplex vertices is within specified tolerance, output optimal set of parameters as $p^*=p_1$.
Reflect: Compute $p_r = p_0 + \eta (p_0-p_{n+1})$ with $\eta >0$. If $J(p_1)\le J(p_r)<J(p_n)$, then $p_{n+1}=p_r$, go to Order.
Expand: If $J(p_r)<J(p_1)$, then $p_e=p_0+\mu (p_r-p_0)$. Further if $J(p_e)<J(p_r)$, then $p_{n+1}=p_e$ and go to Order, else $p_{n+1}=p_r$ and go to Order.
Contract: Here $J(p_r)\ge J(p_n)$, so $p_c=p_0+\rho (p_{n+1}-p_0)$. Further if $J(p_c)<J(p_{n+1})$, then $p_{n+1}=p_c$ and go to Order.
Shrink: Here $J(p_c)>J(p_{n+1})$, replace $p_i=p_1+\sigma (p_i-p_1)$ for all $i\in [2,n+1]$ and go to Order.

Note $\eta ,\mu ,\rho ,\sigma$ are respectively the reflection, expansion, contraction and shrink coefficients and their standard values are $\eta =1,\mu =2,\rho =0.5,\sigma =0.5$. The algorithm terminates with the optimized values of the $SI^2Q^2R^2VD$ parameters *p* that justify the recorded observations.

### Controlled vaccination

Here the aim is to find a model based feedback control strategy that leads to a steady decrease in number of infections by systematically modulating the daily rate of fully vaccinated. The dynamics of the infectious variants can be written in a matrix form as follows

14 $$\begin{aligned} \begin{pmatrix} \dot{I}_1 \\ \dot{I}_2 \end{pmatrix} = \begin{pmatrix} (1-\alpha _f)\frac{\beta _t S}{N}-(\tau +\rho ) &{} 0 \\ 0 &{} (1-\alpha _f)\frac{\beta _t (S+R_1)\beta _v}{N}-(\tau +\rho ) \end{pmatrix} \begin{pmatrix} I_1 \\ I_2 \end{pmatrix} {.} \end{aligned}$$

Now consider a positive semi-definite function *V* equivalent to the energy of the infectious pandemic,

15 $$\begin{aligned} V = (I_1,\; I_2)^T\cdot (I_1,\; I_2) \succcurlyeq 0 \end{aligned}$$

which is positive everywhere in $\mathbb {R}^2$ except at the DFE where it has 0 energy. Therefore, in order to stop the epidemic, a series of control actions must be executed that asymptotically reduce this infectious energy to 0 thus stabilizing at the DFE. This is equivalent to setting the gradient of the energy *V* to be negative semi-definite to obtain,

16 $$\begin{aligned} \dot{V} = (I_1\; I_2) \begin{pmatrix} 2(1-\alpha _f)\frac{\beta _t S}{N}-2(\tau +\rho ) &{} 0 \\ 0 &{} 2(1-\alpha _f)\frac{\beta _t (S+R_1)\beta _v}{N}-2(\tau +\rho ) \end{pmatrix} \begin{pmatrix} I_1 \\ I_2 \end{pmatrix}\preccurlyeq 0 {,} \end{aligned}$$

which is negative everywhere in $\mathbb {R}^2$ except at the DFE where it is 0. The two eigenvalues of the coefficient matrix in (16) appear on its main diagonal and correspond to the dynamics of the primary and the delta variant of the pandemic in the US. In order to enforce the negativity condition on both, the time-varying controlled vaccination rate parameter $\alpha _f$ must be given by (17) that asymptotically stabilizes the pandemic.

17 $$\begin{aligned} \alpha _f \ge \max (1-\frac{(\rho +\tau )N}{\beta _t S},\;1-\frac{(\rho +\tau )N}{\beta _t(S+R_1)\beta _v}) \end{aligned}$$

This ensures the respective rate of reproductions are less than 1 so that the net infections decelerate; if not, they keep rising.

## Results

The COVID-19 pandemic dynamics are modeled by the $SI^2Q^2R^2VD$ system of (1)–(9) as a basis for analyzing the evolution of the two dominant waves in the US largely driven by the dominant variants of SARS-CoV-2. We refer to the large spike in reported cases in January 2021 as the first major wave in the US and the large spike in reported cases in September 2021 as the second major wave in the US. The intervention parameters are learnt from the reported data on daily cases^28^ and used to interpret the two major waves to be driven by the two major variants and the unvaccinated pool of people driving the second surge in infections.

### Model analysis

The baseline transmission rate $\beta _t$ is designed by the following sigmoid function,

18 $$\begin{aligned} \beta _t=((\underline{\beta }+\bar{\beta })+(\bar{\beta }-\underline{\beta })*tanh((t-300)/100))/2 {.} \end{aligned}$$

The rates of fully vaccinated and delta variant are modeled by the following step function,

19 $$\begin{aligned} \alpha _f,\beta _v\in \mathbb R_0^+: \alpha _f=0,\;\forall t<f,\beta _v=0,\;\forall t<v {.} \end{aligned}$$

The nine states of the $SI^2Q^2R^2VD$ system $\{S,I_1,I_2,Q_1,Q_2,R_1,R_2,V,D\}$, with non-negative initial conditions, always evolve with non-negative values and belong to $\mathbb {R}^9_+$. This follows from the property that $\dot{S}\ge 0$ when initial $S^0=0$ in Eq. (1). Similarly $\{\dot{I}_1,\dot{I}_2,\dot{Q}_1,\dot{Q}_2,\dot{R}_1,\dot{R}_2,\dot{V},\dot{D}\}\ge \mathbf {0}$ for their initial values $\{I_1^0,I_2^0,Q_1^0,Q_2^0,R_1^0,R_2^0,V^0,D^0\}=\mathbf {0}$ substituted out their respective differential equations of (2)–(9). Summing equations (1)–(9) yields $\dot{S}+\dot{I}_1,\dot{I}_2,\dot{Q}_1,\dot{Q}_2,\dot{R}_1,\dot{R}_2,\dot{V},\dot{D}=0$, which implies the solutions of the $SI^2Q^2R^2VD$ system are invariant in the set given by $\{S,I_1,I_2,Q_1,Q_2,R_1,R_2,V,D\}\in \mathbb {R}^9_+:S+I_1+I_2+Q_1+Q_2+R_1+R_2+V+D=N$.

**Figure 2:**
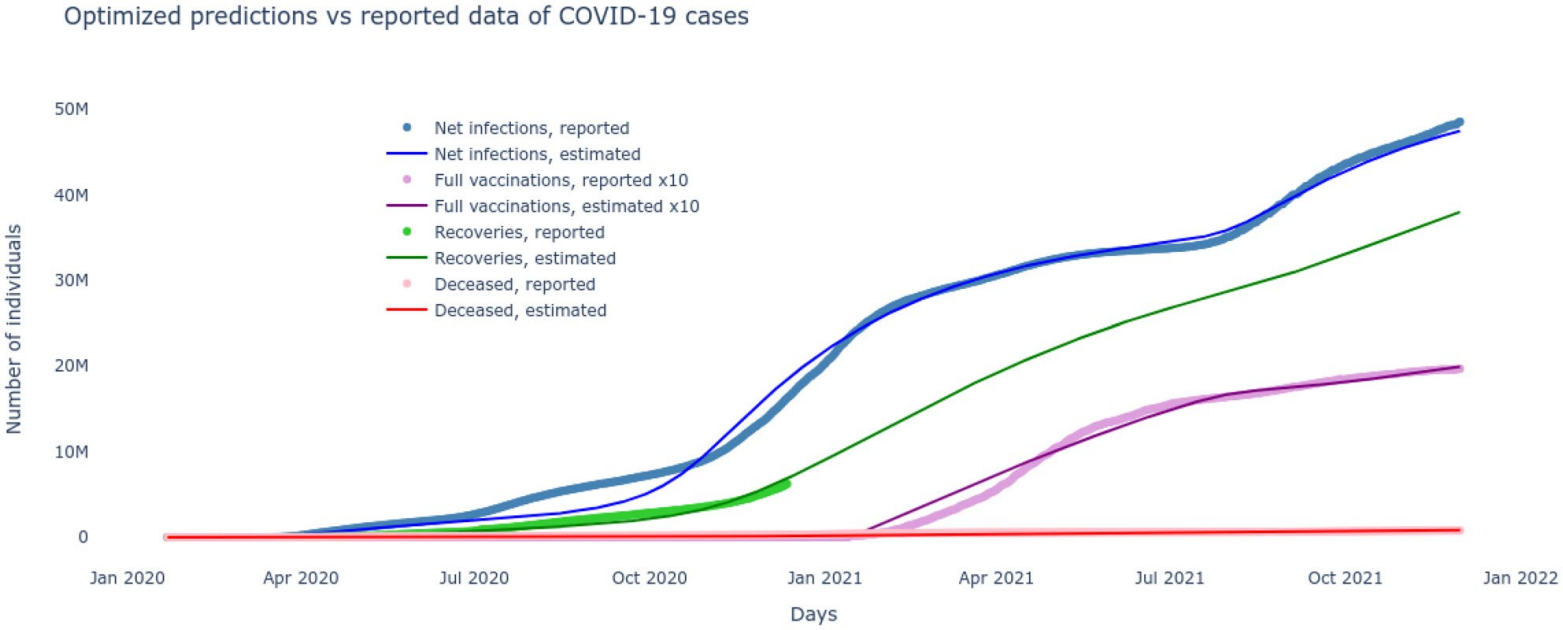
The $SI^2Q^2R^2VD$ system response with optimized parameters $\underline{\beta },\bar{\beta },v,\beta _v,\alpha _f,\tau ,\rho ,\delta$ closely matches the reported COVID-19 data.

Note that although, we have used the same mortality rate for both the variants, distinct ones can be easily incorporated in the model, if necessary.

### System identification

**Figure 3:**
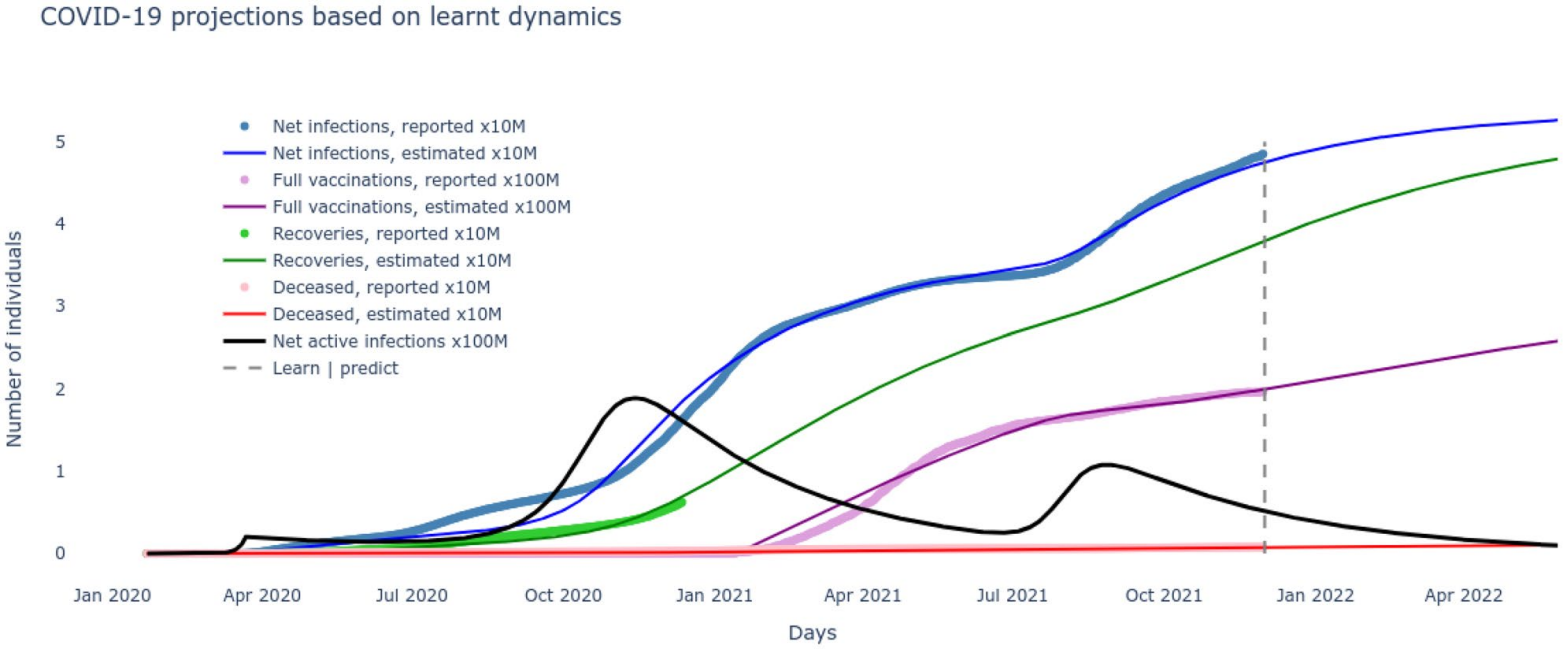
The $SI^2Q^2R^2VD$ system projections with optimized parameters $\underline{\beta },\bar{\beta },v,\beta _v,\alpha _f,\tau ,\rho ,\delta$ learned over historical COVID-19 data.

The unknown parameters of the $SI^2Q^2R^2VD$ system include $\underline{\beta },\bar{\beta },v,\beta _v,\alpha _f,\tau ,\rho ,\delta$ and are to be learned from the publicly available reported cases^28^. Daily cumulative infections and number of recovered, deceased and fully vaccinated between January 22, 2020 to November 30, 2021 are recorded in vectors $I_r,R_r,V_r,D_r$ respectively. Now, the best set of pandemic parameters are the ones for which the $SI^2Q^2R^2VD$ model predictions most closely match the reported cases. The minimization problem of (13) is constrained to the coupled dynamics of the nonlinear $SI^2Q^2R^2VD$ equations (1)–(9), that cannot be integrated analytically, hence neither could the gradients of the cost function be computed. Therefore, a derivative free stochastic optimization technique of Nelder and Mead^29^ is adapted to solve the nonlinear optimization problem. The results plotted in Fig. 2 show a very good match between the $SI^2Q^2R^2VD$ model predictions and the reported data in terms of infected, recovered, vaccinated and deceased. Besides finding the baseline time-varying sigmoid transmission parameters and the test/quarantine, recovery and death rates, it also deduces the start and rate of the second major wave and projects the recovered cases for the rest of 2021 where no data was available.

**Figure 4:**
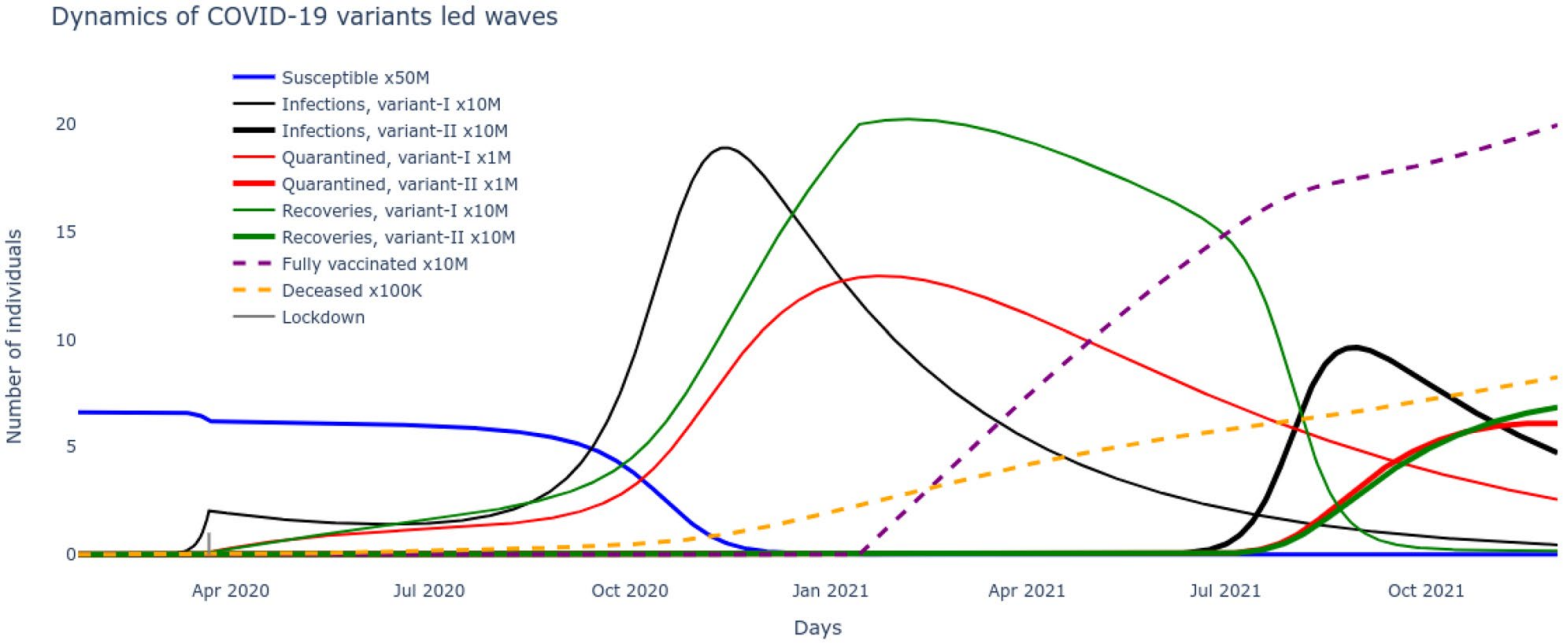
The optimized $SI^2Q^2R^2VD$ system reveals the underlying dynamics of the two major variant-driven waves.

### Analysis of waves and variants

**Figure 5:**
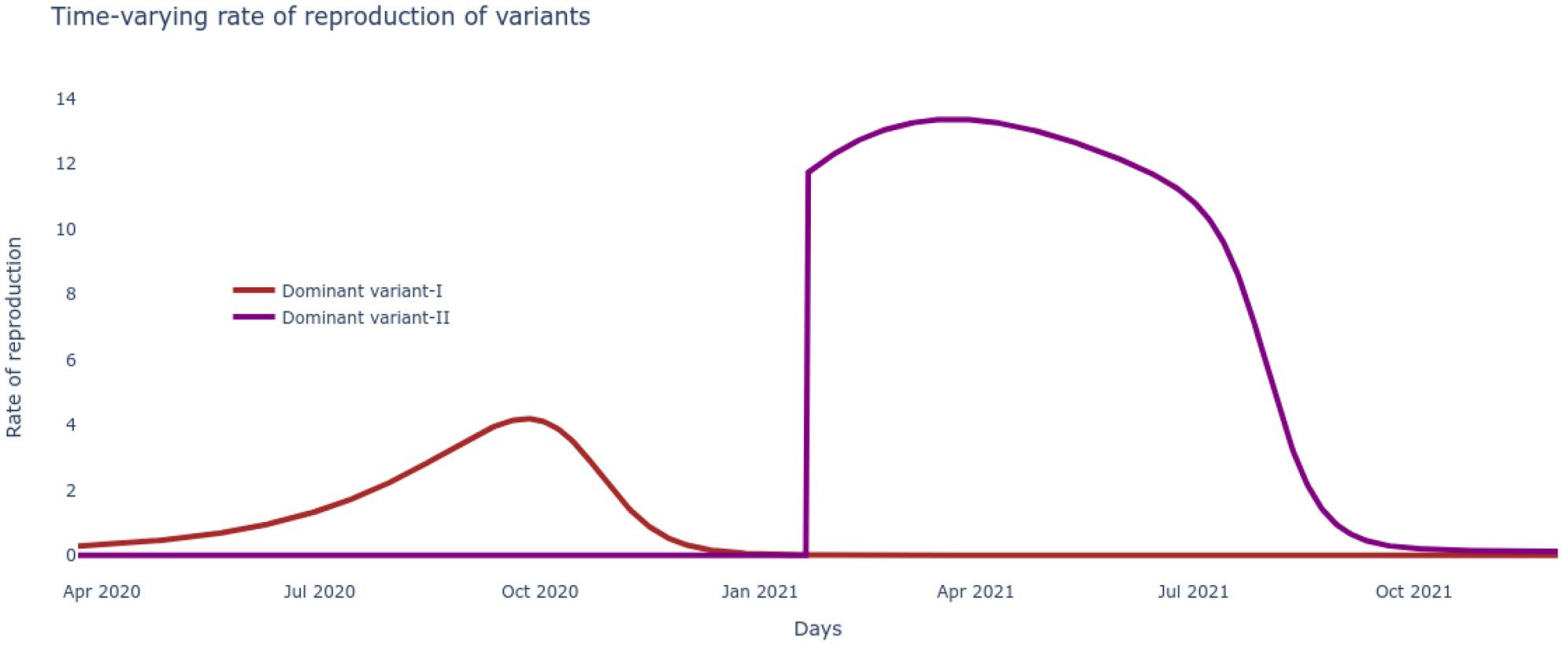
The time-varying rate of reproduction for the two dominant variants reveals a delta wave to be more contagious.

Based on the optimal set of parameters $\underline{\beta },\bar{\beta },v,\beta _v,\alpha _f,\tau ,\rho ,\delta$ learnt for the $SI^2Q^2R^2VD$ system that match the reported data on number of cumulative infections, recovered, vaccinated and deceased, these very variables are now seamlessly projected into the future to track their evolution. These results are plotted in Fig. 3 and give us an immediate sense of the course of the pandemic with the current rates of transmission, recovery and mortality and current rate of interventions of vaccination alongside continuous test and quarantine. Next we want to leverage the development of the $SI^2Q^2R^2VD$ system together with the optimized set of parameters $\underline{\beta },\bar{\beta },v,\beta _v,\alpha _f,\tau ,\rho ,\delta$ to motivate the necessity of the delta variant to drive the second wave. To do so, all the nine states including susceptible, vaccinated, deceased and the two sets of infected, quarantined, recovered of (1)–(9) are solved for by inserting the optimized rates of transmissions, test/quarantine, recovery, vaccination and mortality. The two distinct waves are now clear from the results plotted in Fig. 4. A careful examination reveals that the alpha infectious variant $I_1$ is the driver for the first wave and its delta variant $I_2$ is the driver for the second wave. The reason can be noted from the rate of reproductions of the two variants plotted in Fig. 5, with $\mathcal R_t^0>1$ (alpha variant) during the first wave and $\mathcal R_t^v\gg 1$ (delta variant) during the second wave. Further, the impossibility of the alpha variant causing a second major wave stems from the fact that, beyond the first major wave, the minimum number of susceptible/S are insufficient to cause a surge,

20 $$\begin{aligned} S \ngtr \frac{(\rho +\tau )N}{(1-\alpha _f)\beta _t} \approx 13M {.} \end{aligned}$$

(M stands for million) as evident from the low numbers of S in Fig. 4 during the year 2021. The inequality itself is derived from the condition on reproduction rate (11). Also, the highly contagious nature of the delta variant, widely reported in literature^30^ can be verified through Fig. 5.

### Wave of unvaccinated

**Figure 6:**
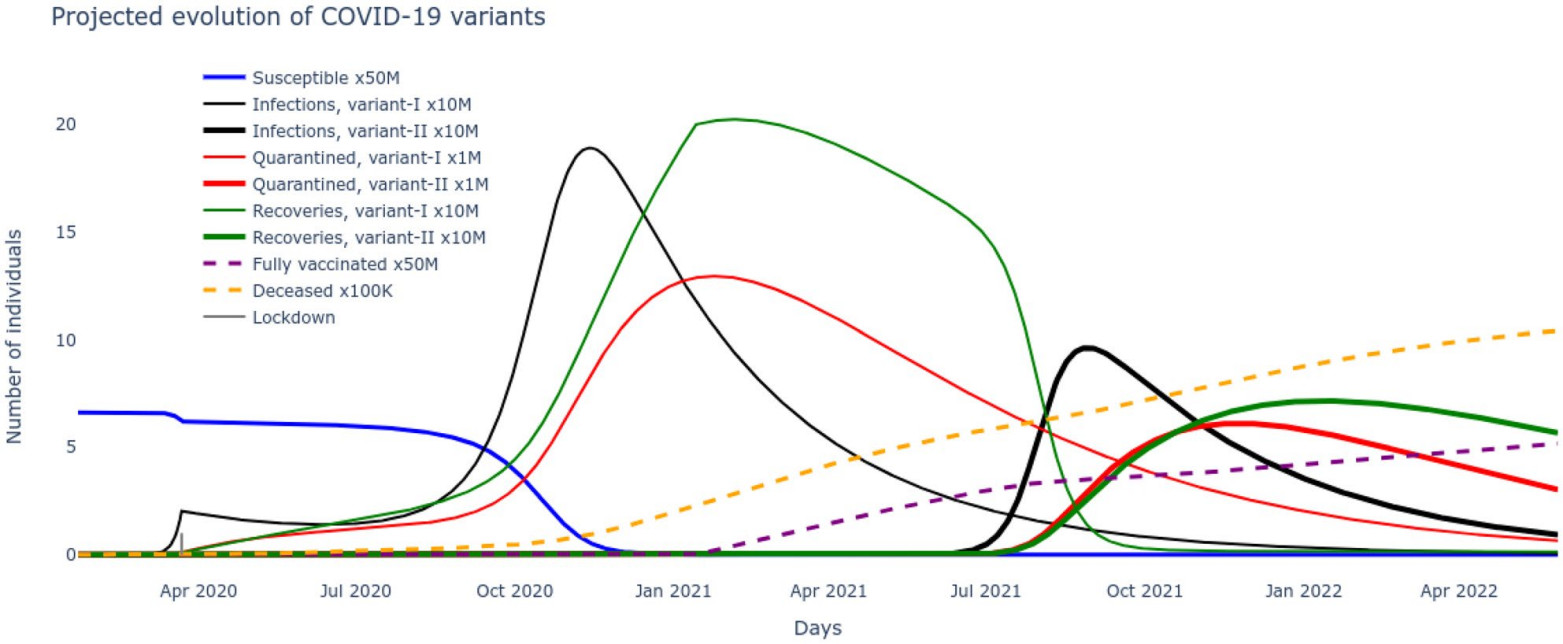
The optimized $SI^2Q^2R^2VD$ system projections reveal the evolution of the variant-driven waves.

Now that we demonstrated the second wave could not have been caused by the alpha strain of SARS-CoV-2 and that another variant, in this case delta was necessary, the next step is to reason out what caused this surge in spite of administration of efficacious vaccines. Note that during the surge of infections causing the second wave, the susceptible/S alone could not have caused it as explained through (20). The only other possibility entails addition of the large pool of people that recovered from the alpha strain but remained unvaccinated i.e. $R_1$, which then satisfies the condition,

21 $$\begin{aligned} S+R_1 \gg \frac{(\rho +\tau )N}{(1-\alpha _f)\beta _t\beta _v} \approx 13M {.} \end{aligned}$$

derived from the condition on variant reproduction rate (12). Indeed, the growth of $R_1$ is supported by the investigation in Fig. 5, which follows from the flow in Fig. 1. However, an increase in the rate of vaccination uptake would certainly have reduced the $R_1$ pool, pushing them to fully vaccinated/V pool, thereby decreasing variant reproduction rate $\mathcal R_t^v$ and its corresponding surge. Finally the optimized set of pandemic parameters $\underline{\beta },\bar{\beta },v,\beta _v,\alpha _f,\tau ,\rho ,\delta$ can be used to project the possible course of the pandemic in the US by integrating the $SI^2Q^2R^2VD$ system of (1)–(9). The results are plotted in Fig. 6.

Note that this analysis also justifies the usage of same rate of infection by the second variant of susceptible and those that recovered from the first strain, as the number of recovered gradually outnumber the susceptible; therefore, adding another parameter may just further complicate the optimization problem.

## Discussion

The $SI^2Q^2R^2VD$ system projections based on optimized parameters $\underline{\beta },\bar{\beta },v,\beta _v,\alpha _f,\tau ,\rho ,\delta$ obtained by derivative-free stochastic optimization matched well with the recorded data as seen in Figs. 2 and 3. Further, with this as basis, two crucial properties: the necessity of the two major variants driving the two major waves and the growing population of recovered from the alpha strain who remained unvaccinated driving the second surge in infections were proven through Eqs. (20)–(21) and supported by Figs. 4 and 5. The very high number of mortality caused by the the major waves are evident from Fig. 6 and is a matter of grave concern. The question naturally arises, if it was possible to suppress the second wave in particular, thereby reducing the fatalities, by systematically modulating the vaccine administration.

**Figure 7:**
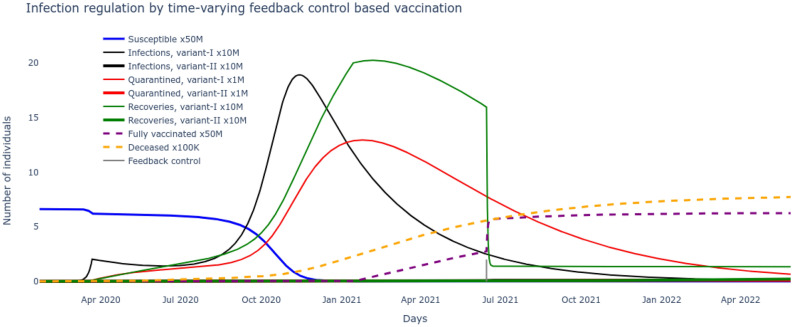
Eliminating the delta wave by time-varying feedback control based vaccination.

The answer lies in systematically regulating the daily rate of fully vaccinated by using the principles of feedback control. A time-varying control law $\alpha _f$ is derived in (17), that guarantees a negative gradient of the infectious energy of the pandemic, asymptotically leading to disease free equilibrium/DFE. During the first major wave, $\mathcal R_t^0$ corresponding to the alpha strain will dominate and during the second major wave $\mathcal R_t^v$ corresponding to the delta strain will dominate.

In the first scenario, this control law $\alpha _f$ is applied to the $SI^2Q^2R^2VD$ pandemic system in the early stages of the second wave (July 2021), reacting to the widely detected emergence of the delta variant, when vaccines were widely made available. From the results plotted in Fig. 7, one can conclude that the second wave could be largely avoided leading to a substantial decrease in mortality (reduction by 3M). The time-varying control of $\alpha _f$ suggests a higher percolation of rate of fully vaccinated/V as a necessary step towards attaining the target of suppressing the second wave.

In the next scenario, a time-invariant controlled vaccination rate parameter $\alpha _f$ can be derived from (17) that considers the supremum of the time-varying policies to give (22) where $\beta _v\ge 1$ and $\bar{\beta }$ corresponds to maximum transmission with no social restrictions.

22 $$\begin{aligned} \alpha _f =1-\frac{(\rho +\tau )}{\bar{\beta }\beta _v} \end{aligned}$$

**Figure 8:**
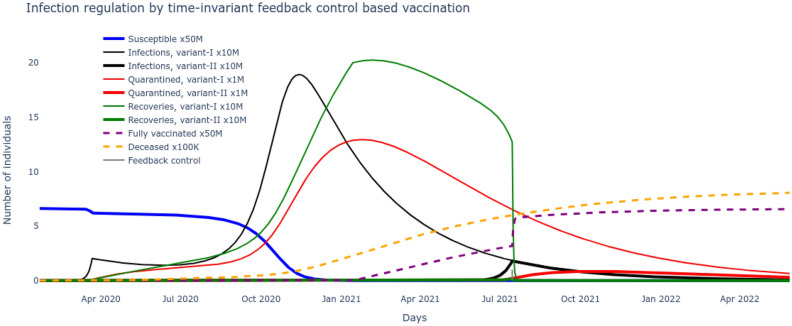
Suppressing the delta wave by time-invariant feedback control based vaccination.

The results plotted in Fig. 8 reveal that this feedback control based vaccination approach, even with a delayed application (from August 2021), could suppress the peak of the second wave leading to reduced mortality (reduction by 2M). A comparison of Figs. 7 with 8 reveals the importance of timing of adopting feedback control based vaccination policy i.e. early application can suppress the pandemic wave more effectively and save many more lives.

## Data Availability

All data generated in this study are have been included in this paper.
